# Systemic Inflammatory Response and Atherosclerosis: The Paradigm of Chronic Inflammatory Rheumatic Diseases

**DOI:** 10.3390/ijms19071890

**Published:** 2018-06-27

**Authors:** Aikaterini Arida, Athanasios D. Protogerou, George D. Kitas, Petros P. Sfikakis

**Affiliations:** First Department of Propaedeutic and Internal Medicine and Joint Rheumatology Program, National and Kapodistrian University of Athens Medical School, GR-115 27 Athens, Greece; athanprot@gmail.com (A.D.P.); george.kitas@nhs.net (G.D.K.); psfikakis@med.uoa.gr (P.P.S.)

**Keywords:** atherosclerosis, cardiovascular disease, inflammation, rheumatoid arthritis, lupus, ankylosing spondylitis, psoriatic arthritis

## Abstract

Patients with Chronic Inflammatory Rheumatic diseases (CIRD) are at increased risk of cardiovascular disease (CVD), ascribed not only to classical risk factors, but also to the presence of chronic systemic inflammatory response. Αtherosclerosis, the cornerstone of CVD, is known to be accelerated in CIRD; rheumatoid arthritis promotes atheromatosis and associates with preclinical atherosclerosis equivalent to Diabetes Mellitus, which also seems to apply for systemic lupus erythematosus. Data on ankylosing spondylitis and psoriatic arthritis, albeit more limited, also support an increased CV risk in these patients. The association between inflammation and atherosclerosis, has been thoroughly investigated in the last three decades and the role of inflammation in the pathogenesis and progression of atherogenesis has been well established. Endothelial dysfunction, oxidative stress in vascular endothelial cells and macrophage accumulation, toll-like receptor signaling, NLPR-3 formation and subsequent pro-inflammatory cytokine production, such as TNFa, IL-1β, IL-6, and TNF-like cytokine 1A, are few of the mechanisms implicated in the atherogenic process. Moreover, there is evidence that anti-inflammatory biologic drugs, such as anti-TNF and anti-IL1β agents, can decelerate the atherogenic process, thus setting new therapeutic targets for early and effective disease control and suppression of inflammation, in addition to aggressive management of classical CV risk factors.

## 1. Introduction

**Figure 1:**
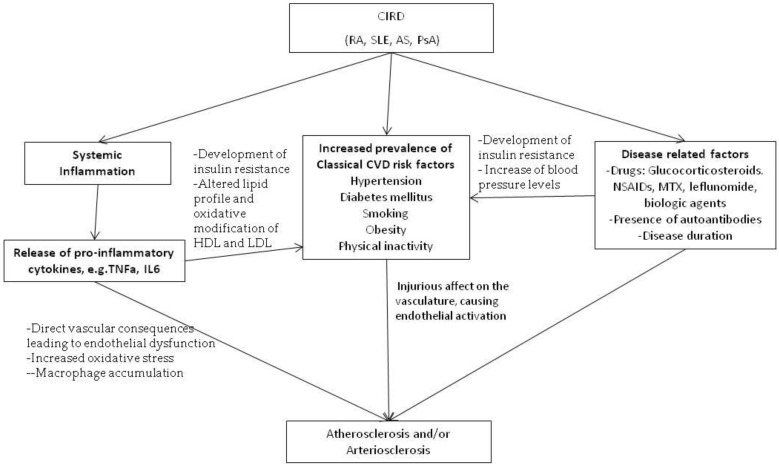
Risk factors involved in the pathogenesis of atherosclerosis and/or arteriosclerosis in chronic inflammatory rheumatic diseases.

Chronic Inflammatory Rheumatic diseases (CIRD), such as Rheumatoid arthritis (RA), systemic lupus erythematosus (SLE), and seronegative SpA, are associated with high prevalence of cardiovascular disease (CVD), leading to increased morbidity and mortality in patients [1,2,3,4]. In these populations, accelerated atherosclerosis, the cornerstone of CVD, is ascribed not only to classical CVD risk factors such as arterial hypertension, glucose intolerance, sedentary life, smoking and dyslipidemia, which are in several cases more prevalent in patients with CIRD, but also to the presence of chronic inflammation and perhaps disease-related therapies as well [1,5,6,7] (Figure 1).

Rheumatoid arthritis patients are known to have an increased risk of CVD compared to controls, equivalent to that of diabetes mellitus (DM). Moreover, given that existing CV risk assessment models used for the general population underestimate the CVD risk in RA, in 2009 the EULAR task force recommended that the CV risk estimate should be multiplied by 1.5 when certain disease characteristics are present, and this was subsequently carried over in the updated EULAR recommendations in 2017 for all patients with RA [1,4,8,9,10]. Similarly, patients with SLE and/or antiphospholipid syndrome (APS) have been shown to have increased—at least 2- to 3-fold—mortality due to CVD events compared to the general population, which is not fully explained by classical risk factors included in the Framingham risk equation [11,12,13]. Studies examining subclinical CVD in SLE and APS patients reveal higher carotid intima-media thickness (cIMT) and the increased prevalence of atherosclerotic plaques, almost 2.5-fold compared to healthy controls [14,15,16]. As in RA, increased inflammatory burden, as well as immune dysregulation are thought to play a crucial role in plaque progression and promotion of CVD in these patients. Even though data is more limited, expanding evidence suggest an increased CVD morbidity and mortality in patients with ankylosing spondylitis (AS) and psoriatic arthritis (PsA), although the more frequent use of NSAIDs could contribute to this increase [4,17,18,19,20,21,22]. Interestingly, effective disease control and effective suppression of inflammation in these patients associates with less accelerated atherosclerosis, once again indicating that chronic inflammation has a detrimental effect on endothelial function, atherogenesis, and arterial stiffening (arteriosclerosis) [23,24].

The causal relationship between atherosclerosis and inflammation has been the subject of extensive investigation in recent years. Systemic markers of inflammation, such as C-reactive protein (CRP) and the proinflammatory cytokines TNFa and IL-6, are known to independently predict CVD events [25,26] and the atherosclerotic process can be modulated by anti-inflammatory molecules targeting different inflammatory pathways [27]. Several lines of evidence suggest that CVD, from endothelial damage and atherogenesis to plaque rupture, is an immune-mediated disease and many studies have focused on determining the exact mechanisms involved in the atherosclerotic process. Alteration of lipoprotein concentrations, oxidative stress, and macrophage accumulation, as well as endothelial injury and dysfunction, activation of the innate immune system, and increased circulating cytokines are just a few of the underlying pathogenic mechanisms. Similarities of chronic inflammatory processes and the dysregulated immune responses seen in CVD and CIRD further support this association [28].

In the present narrative review, we summarize current knowledge regarding the prevalence of clinical and subclinical CVD in patients with RA, SLE, AS, and PsA and the common pathogenetic background between atherosclerosis and these CIRD, focusing to the underlying complex mechanisms of inflammation and immunity. We did not consider sclerodermia due to the very different nature of the disease and the different way in which it causes CVD. Regarding polymyalgia rheumatoca, a considerable difficulty is the relatively older age of the patients as well as the limited bibliography on the subject. Similarly, data on Sjogren’s syndrome is very limited.

## 2. Search Strategy

We searched the Medline/Pubmed database for primary articles published through March 2018 on the association of CVD and CIRD, namely rheumatoid arthritis, systemic lupus erythematosus, ankylosing spondylitis, and psoriatic arthritis. More specifically, we included studies examining (a) the prevalence of CV events or subclinical CVD (endothelial dysfunction, arterial stiffness, arterial wall hypertrophy or atheromatosis), (b) the pathogenesis of atherosclerosis in CIRD in terms of the burden of chronic inflammation and immune dysregulation, as well as (c) studies reporting on the management of atherosclerosis via anti-inflammatory drugs. Search terms included: atherosclerosis, cardiovascular disease, intima-media thickness (IMT), pulse wave velocity (PWV), and atheromatic plaques in combination with inflammation, rheumatoid arthritis, lupus, ankylosing spondylitis, and psoriatic arthritis. We included prospective and retrospective, experimental or observational studies, systematic reviews, and meta-analyses, whereas comments were excluded. Abstracts presented in conferences were not considered.

## 3. Thematic Sections

### 3.1. Epidemiology

#### 3.1.1. Rheumatoid Arthritis

The association of CVD and RA has been widely investigated in the past years and it is now well established that RA patients have an excess risk for developing CVD, comparable to that reported for patients with Diabetes Mellitus [8,9,29]. The increased prevalence of CVD refers to all components of atherosclerosis, from endothelial dysfunction and arterial stiffness to abnormal morphology and the formation of atheromatic plaques and CV events, namely myocardial infarction (MI) and stroke. A meta-analysis by Levy et al. concluded that RA patients present an excess risk of fatal MI compared to the general population and this risk was found to be similar in RA patients from a Danish cohort to that of patients with DM [10,30]. This was later verified for MI in a more recent meta-analysis [17] as well as for stroke in another meta-analysis involving 16 studies [31] (Table 1).

Ambrosino et al. in a meta-analysis concluded that RA patients have increased Pulse wave velocity (PWV) and Augmentation index (Aix) compared to controls and that alteration, present even in early-stage disease, was associated with the severity of the inflammatory status [40]. The same researchers showed that RA is associated with higher carotid intima-media thickness (cIMT) and increased presence of carotid atheromatic plaques [38]. Similarly, accelerated femoral atheromatosis was found to be analogous to DM in in a study by Protogerou et al. [52]. IMT progression in RA was associated with systemic inflammation and classical CVD risk factors in a study by del Rincon et al. [53], however, CVD progression was decelerated when RA disease inflammation was suppressed, indicating that chronic systemic inflammation alone has a substantial impact on promoting arterial disease in RA [54,55,56]. This is further supported by the fact that increased atherogenesis in RA has been shown to be independent of the presence of classical risk factors [57,58]. Finally, data from two recent meta-analyses found that endothelial function estimated by flow-mediated dilation (FMD)—considered as an independent predictor of CV events—is also impaired in patients with RA [48,49] (Table 1).

#### 3.1.2. Systemic Lupus Erythematosus and Antiphospholipid Syndrome

The range of cardiovascular involvement in SLE includes atherosclerosis, vasculitis, Raynaud’s phenomenon, and a pro-coagulant tendency associated with the presence of antiphospholipid antibodies. A recent meta-analysis found strong epidemiologic evidence of at least 2- to 3-fold elevated risks of a CVD event in patients with lupus compared to the general population, and this risk was alarmingly elevated in young women [13]. Paradoxically, while the risk of deaths related to lupus activity has decreased over time, the risk of deaths due to circulatory disease does not appear to have diminished [59] and this is further supported in a recent study by Tektonidou et al. by the fact that hospitalization of SLE patients for acute MI or stroke has not decreased [60] (Table 1).

Concerning subclinical atherosclerosis, Wu et al. in a meta-analysis including 80 studies that examined cIMT and carotid plaques, concluded that both indices were significantly increased compared to controls [14]. This was confirmed in a later meta-analysis, which also showed impaired FMD in SLE patients compared to controls [16], and further data support that FMD in SLE could serve as a marker of atherosclerotic vascular complications [61,62]. In addition, a study from our center revealed that the risk of atherosclerotic plaques in carotid, as well as femoral beds was comparable to that of DM and rheumatoid arthritis [15]. Similarly, arterial stiffness as measured by PWV and AIx has been proven to be increased in SLE patients compared to controls [41,42,43] and this is related to both traditional risk factors—especially metabolic syndrome and age—and SLE-related risk factors, such as disease activity measured by SLEDAI, SLICC damage index, higher C3 levels, presence of ds-DNA antibodies, and glucocorticoid therapy [41,42,43,50,51,63,64] (Table 1).

#### 3.1.3. Ankylosing Spondylitis

CVD results in increased mortality in AS, reaching a hazard ratio of 1.8 [24,32,33,34]. Studies have reported increased risk of acute coronary syndromes and stroke in patients with AS compared to controls and this was verified in a recent meta-analysis involving 18 studies [18,36,65,66]. Regarding subclinical atherosclerosis, our recent meta-analysis of 12 controlled studies found an increased overall IMT compared with matched controls, however, this was not evident in studies involving patients with low disease activity (mean BASDAI < 4) or in those studies that included >50% of patients treated with anti-TNF [23]. Interestingly, overall data from five studies revealed that carotid plaque burden was not increased in AS patients compared to controls. In the same study, common carotid artery stiffness as well as aortic stiffness were similar between cases and controls, in contrast to previous studies where aortic elasticity and endothelial dysfunction, estimated by PWV and FMD respectively, were impaired [23,44,45,67]. Again, arterial stiffness measured by Aix, was associated with C-reactive protein and AS disease activity score (ASDAS), supporting that disease activity is related to future risk of cardiovascular disease in patients with AS [68] (Table 1).

PsA patients are also considered to be of increased risk of cardiovascular morbidity and mortality compared with the general population [54]. Studies examining CVD in PsA found increased prevalence of CV events in PsA [20,21,37] and another study demonstrated that the prevalence of CVD in PsA resembles the CV risk of RA and the risk of CVD is associated with disease severity and disability index [35]. In a recent meta-analysis, the pooled relative risk of MI was 1.41 and it remained significantly increased after adjusting for traditional CVD risk factors [17], which could indicate the direct impact of PsA per se on the acceleration of CVD. Subclinical atherosclerosis has also been found to be increased in PsA. A recent meta-analysis by Di Minno et al. examining cardiovascular risk markers showed that carotid IMT, FMD, as well as atheromatic plaque index are higher in PsA patients than matched controls [39] and in some cases this was correlated positively with disease severity and negatively with the use of TNF-a blockers [69]. Carotid IMT and FMD were impaired even in the absence of classical risk factors in two small studies by Gonzalez-Juanatey et al. [70,71]. This was also the case in a study examining arterial stiffness by PWV in PsA patients without CV risk factors, which has also been reported to be increased in PsA [46,47] (Table 1).

### 3.2. Classical Cardiovascular Risk Factors and Metabolic Syndrome (MetS)

Inflammation can be a risk factor for developing MetS, as it can influence metabolic homeostasis. Metabolic syndrome can transiently occur in lean individuals during infection, where increased secretion of TNF, IL-6 and IL-1 by macrophages induces a temporary insulin-resistant state [72]. Regarding traditional risk factors such as hypertension, DM, smoking, hypercholesterolemia, obesity, and physical inactivity, they are commonly present in RA and this is likely to explain at least some of the excess CV risk in these patients [73]. RA patients have high prevalence of arterial hypertension, ascribed partially to the use of certain antirheumatic drugs such as corticosteroids, NSAIDs, cyclosporine, and leflunomide [74,75,76,77]. Interestingly, an easily modifiable risk factor such as arterial hypertension is not only under-diagnosed in RA, but also under-treated, further adding to the already excessive burden for CVD [78,79]. The increased presence of metabolic syndrome (MetS) in RA compared to controls, especially insulin resistance, is associated with disease activity and higher inflammatory markers, suggesting that inflammatory processes play a notable role in this case [80,81,82] (Table 1).

Several studies have shown that TNFa and IL-6 are involved in the development of insulin resistance [83,84] and blocking of TNF-alpha activity with TNFa antagonists results in improved insulin sensitivity [85,86]. Moreover, the use of methotrexate independently correlates with a reduced propensity to MetS, whereas long-term glucocorticoid exposure does not seem to associate with a higher prevalence of the metabolic syndrome [87,88]. Of particular interest is the ‘lipid paradox’ of active RA, where the presence of excessive inflammatory burden leads to a decrease of total cholesterol, high-density lipoprotein (HDL) cholesterol, and low-density lipoprotein (LDL) cholesterol, while their CVD risk is elevated [89,90,91] (Table 1).

Moreover, chronic inflammation leads to oxidative changes that alter the HDL structure, causing an impairment to the normal anti-inflammatory, antioxidant, and cardioprotective function of HDL [92]. Paradoxically, anti-inflammatory therapies, especially TNFa inhibitors and methotrexate, coincide with an increase in an overall increase of lipid components, but mostly HDL, which improves the TC/HDL ratio in patients with RA, and this lipid profile is associated with a reduction in the number of CV events, which is probably due to the anti-inflammatory effect and subsequent suppression of RA-associated inflammation [93,94,95]. The use of statins and n-3 fatty acids, apart from ameliorating the lipid profile and reducing the atherosclerotic burden, has anti-inflammatory properties that may result in an even greater CVD risk reduction [96,97,98] (Table 1).

Finally, the impaired physical activity in RA patients may also affect the risk of CVD, since low physical activity in RA women is associated with increased levels of oxidized low-density lipoprotein (oxLDL) and insulin, with reduced levels of HDL and atheroprotective antibodies against phosphorylcholine, and with insulin resistance [99]. Among traditional risk factors, smoking is not only an independent risk factor of atherosclerosis, but has also been associated with increased susceptibility to and worse prognosis of RA in individuals carrying shared epitope alleles and producing anti-citrullinatedprotein/peptide antibodies (ACPA) [100,101].

In SLE, insulin resistance (IR) is more prevalent than in controls and MetS was found to be associated with Body Mass Index (BMI) and higher levels of inflammation rather than lupus disease activity, damage scores, or the use of corticosteroids and antimalarial drugs [102]. Compared to RA, IR index is lower in SLE and the mechanisms underlying IR seem to differ between the two inflammatory diseases. Obesity seems to be an important factor in developing IR in SLE rather than in RA and IR in SLE is not associated with TNFa and IL-6 levels, as is in RA. Moreover, patients with SLE have higher concentrations of Lp(a), which is known to be associated to IR in some populations, than those with RA [103,104]. In addition, SLE patients have a tendency to develop pro-atherogenic lipids, which are aggravated by disease activity [105], as they show increased total cholesterol and triglycerides, decreased levels of normal HDL but a greater proportion of proinflammatory HDLs, and oxidized LDL (oxLDL) levels compared to healthy controls [106,107]. Since the use of statins seems to have a therapeutic effect not only on lipids, but also on fasting glucose and on CRP levels, control of dyslipidemia with statins in these patients could be of great importance (Table 1).

Although data is more limited, MetS prevalence has been found to be high in patients with AS and, as expected, was associated with disease activity, suggesting an association with the high inflammatory burden of the disease. More specifically, several studies, as well as a meta-analysis of 15 case-control studies, have shown that patients with AS have higher blood pressure levels, lower HDL, and triglyceride levels, are significantly more often smokers and, therefore, have higher atherogenic indices compared to controls [19,108,109]. This is compatible with current knowledge, since evidence supports a link between inflammatory cytokines such as TNFa, which is implicated in AS pathogenesis, and key components of HDL homeostasis and therefore an altered lipid profile. Regarding PsA, data suggests an even worse metabolic profile than that reported for RA or AS, with higher prevalence of impaired fasting glucose, low HDL cholesterol, high triglycerides level, central obesity, and high blood pressure compared to the other two diseases [21,110]. Again, correlation with disease activity [111] points to an adverse impact of high grade inflammation and particularly elevated TNFa levels, and data on the therapeutic use of biologic agents suggests a positive effect of TNFa inhibitors not only on articular and cutaneous symptoms, but also on the metabolic profile of these patients [112]. Data on the improvement of HDL function after successful psoriasis treatment [113] could also apply for PsA (Table 1).

## 4. Immunological Mechanisms

### 4.1. Endothelial Dysfunction

Endothelial dysfunction is a necessary condition for the development of atherosclerosis. The endothelium is the key regulator of vascular homeostasis and low oxidative stress, as it is able to respond to physical and chemical signals by the production of a wide range of factors that regulate vascular tone, cellular adhesion, thromboresistance, smooth muscle cell proliferation, and vessel wall inflammation. The endothelium modulates vasomotion, by release of vasodilator substances, namely as nitric oxide (NO) and prostacyclin (PGI2), as well as via vasoconstrictor agents, such as endothelin, and via the conversion of angiotensin I to angiotensin II at the endothelial surface. Moreover, NO normally maintains the vascular wall in a quiescent state by inhibition of inflammation, cellular proliferation, and thrombosis.

Endothelial dysfunction is basically the maladaptive response of endothelial cells to pathological stimuli, mechanical or chemical. This results in endothelial activation; i.e., upregulated expression of cellular adhesion molecules (intercellular adhension molecule (ICAM)-1), ICAM-3, vascular endothelial adhension molecule (VCAM-1) at the endothelium favoring plaque formation, increased leukocyte diapedesis, increased vascular smooth muscle tone due to impaired processing of vasodilator substances, particularly NO, as well as increased production of vasoconstrictor substances, resistance to thrombosis via platelet aggregation, and oxidative stress upregulation [28,114,115]. Compromised barrier function, as a result of endothelial cell damage, leads to an increase in permeability to lipoproteins and plasma constituents, resulting in penetration of lipids into the arterial wall and subendothelial lipoprotein retention. These retained lipoproteins are subsequently taken up by macrophages to form foam cells and fatty streaks within the vessel wall [116]. Moreover, endothelial dysfunction leads to the accumulation of monocytes and proliferation of smooth muscle cells, which migrate to the lesion and lead to the thickening of the vessel wall and formation of fibrous tissue. Ultimately, a fibrous cap develops over the plaque, which, when becoming unstable, leads to rupture and subsequent thrombosis and CV events [117].

Endothelial dysfunction, while part of a normal immune system defense, can lead to atherogenesis and CV events as a result of prolonged and more intense inflammatory stimuli, which induces sustained endothelial activation. Both traditional and novel cardiovascular risk factors including smoking, aging, hypercholesterolemia, hypertension, hyperglycemia, as well as obesity, elevated CRP, and chronic systemic infection, are all associated with alteration in endothelial function [118]. As previously mentioned, in CIRD, classical risk factors are more prevalent and therefore have a more injurious effect on the vasculature. Moreover, autoimmune-inflammatory mechanisms include the accumulation of inflammatory molecules (lymphocytes and macrophages), presence of autoantibodies, and the secretion of pro-inflammatory cytokines, chemokines and adhesion molecules, which have systemic vascular consequences and subsequently reduce synthesis of NO, initiating the cascade of events leading to endothelial dysfunction and CVD [117,119]. Several studies have focused recently on the correlation between such biomarkers indicative of endothelial dysfunction and CVD in patients with CIRD, particularly RA and on how biologic therapy influences these markers associated with the development of CVD. Dessein et al. showed that serum levels of VCAM-1 were associated with common cIMT and plaque presence and IL-6 was independently related to endothelial activation [115,120,121]. VCAM-1 was also partially correlated with cIMT in RA patients undergoing Infliximab therapy, but this was not evident for carotid plaques or other biomarkers of endothelial cell activation, even though anti-TNF treatment seems to reduce the serum levels of ICAM-3 and P-selectin molecules [122,123]. In another study by Kerekes et al., impaired endothelial function, measured by flow-mediated dilation (FMD), was associated with IFN-γ levels in RA patients [124]. Asymmetric dimethylarginine (ADMA) levels—an inhibitor of nitric oxide (NO) synthase and a possible marker of endothelial dysfunction—are found elevated in RA patients, and this was associated with increased inflammatory markers, even though there seems to be no correlation with in vivo assessments of vascular function and morphology [115,125]. Regarding SLE, data is more limited, but studies have shown increased levels of certain soluble endothelial damage biomarkers, such as ICAM-1 and thrombomodulin (TM) in SLE women and VCAM-1 in patients with lupus nephritis [126,127] Similarly, AS studies indicate that increased levels of ICAM-1, TM and IL-6, as well as increased ADMA serum concentrations are not correlated however with disease activity [128,129,130]. In PsA, both ICAM-1 and VCAM-1 levels have been found to be increased compared to controls and ICAM and IL6 were correlated with Cimt [131,132].

#### 4.1.1. Oxidative Stress

Oxidative stress reflects the imbalance between the production of reactive oxygen species (ROS) and impaired antioxidant capability, which in turn causes cell injury by directly oxidizing cellular protein, lipid, and DNA or via cell death signaling pathways. Oxidative stress has been demonstrated to play an important role in the pathogenesis of atherosclerosis especially by promoting the oxidative modification of LDL. Oxidized LDL takes part in many phases of atherogenesis: stimulates the binding of monocytes to the endothelium, foam cell formation, the development of plaques, plaque destabilization, and thrombotic complications [28,133].

Oxidative stress and inflammation are interrelated and this interaction promotes plaque formation and rupture. Increased oxidative stress is thought to play a role in the pathophysiology of inflammatory diseases such as RA and SLE, contributing to immune system dysregulation and autoimmunity [119,134]. In RA, the overproduction of TNFa is a main contributor to increased ROS release, and this is related to disease activity. ROS conserves oxidative stress, further promoting in this way cell damage and atherogenesis [135,136]. Moreover, oxidative modification of LDL has been linked to TNFa action and HDL constituents may be altered by the inflammation, thus losing their ability to remove cholesterol from atherosclerotic lesions and reducing their antioxidant activity. As previously mentioned, in SLE there is a decrease in normal HDL and a greater proportion of proinflammatory HDLs, which impairs the ability to prevent LDL oxidation [107]. Similarly, studies showed increased levels of oxidative stress markers with AS [137,138] and PsA [139], however, data is limited. Interestingly, control of disease activity via the use of TNFa inhibitors in RA can reduce oxidative stress [140], probably also directly reducing in this way the risk of CVD.

#### 4.1.2. Innate Immunity, Toll Like Receptor (TLR) Signaling, and NLRP3 Inflammasome Activation

Innate immune mechanisms play a central role in atherogenesis, involving the activation of pattern-recognition receptors (PRRs), especially damage-associated molecular patterns (DAMPs), on the surface of ECs, and the induction of inflammatory processes and atherogenesis [141]. Scavenger receptors expressed by macrophages recognize specific epitopes of oxidized LDL and mediate clearance of lipoproteins and intracellular cholesterol accumulation, thereby promoting foam cell formation.

Signaling through the Toll like receptor (TLR) pathway has been implicated in the pathogenesis of autoimmune diseases, including RA, SLE and SpA, as well as in atherosclerosis. TLRs are membrane glycoprotein PRRs that recognize both PAMPs and DAMPs and initiate complex signal transduction pathways that mediate strong inflammatory responses. They are widely expressed on many cell types such as ECs, macrophages, dendritic cells, lymphocytes, and vascular smooth muscle cells, all of which are implicated in atherosclerotic lesion development. Atherosclerotic lesions display enhanced expression of specific TLRs, especially TLR2 and TLR4, on the surface of ECs. In atherosclerosis, an oxidized LDL can trigger TLR signaling, thus mediating macrophage accumulation and infiltration, induction of proinflammatory cytokines, activation of inflammatory cells, and decreased presence of regulatory T cells in the atherosclerotic lesions, conditions which are known to promote atherogenesis [117,142,143].

In RA, there is the pathogenic expression of inflammatory cytokines- including TNF-α, IL-1, and IL-6-by synovial macrophages and an increasing body of evidence supports the role of TLRs in the persistent, progressive activation of macrophages. Several studies have shown an increased expression of different TLRs by cells within the RA joint, as well as an increased responsiveness of RA synovial fibroblasts and RA synovial macrophages to microbial TLR ligands [144,145,146]. Therefore, both RA and atherosclerosis are characterized by chronic inflammation, the accumulation of macrophages, dendritic cells, and B and T lymphocytes caused by the local expression of TLRs and potential endogenous TLR ligands. Moreover, the release of endogenous TLR ligands as well as cytokines, such as TNFa and IL-6, from the inflamed synovial tissue might further activate macrophages in the atherosclerotic plaque and partly explain the increased occurrence and severity of atherosclerosis in RA [144].

In SLE, evidence suggests that the TLR system enhances the expression of pathogenic autoantibodies and possibly contributes to the IFN-α signature pattern that is characteristic of patients with active SLE [147]. More specifically, the release of IFN-α from plasmacytoid dendritic cells in patients with lupus may be mediated by the activation of TLR7 and TLR9 by endogenous RNA and DNA. Further, the release of IFN-α systemically may also promote the activation of macrophages in the atherosclerotic plaques by endogenous TLR2 and TLR4 ligands that are expressed locally. It is also possible that in patients with lupus and atherosclerosis, the plaque dendritic cells may also become activated by coming into contact with RNA- or DNA-containing immune complexes [144].

Regarding AS and PsA, emerging evidence indicates that TLRs play a potential role in the pathogenesis of SpA, which is supported by the fact that certain TLRs have been found to be overexpressed in patients with AS and PsA. De Rycke et al. showed increased TLR4 expression in peripheral blood mononuclear cells from patients with AS, as well as increased TLR2 and -4 expression in the inflamed synovium of patients with SpA (including PsA, AS and undifferentiated spondyloarthritis) compared with osteoarthritis or RA synovium, which was sharply reduced by TNFa blockade [148]. Similarly, Assassi et al. showed the upregulation of gene expression of TLR4 and 5 in AS but not in healthy controls or systemic lupus erythematosus patients and Candia et al. found the increased expression of TLR2 in immature dendritic cells in patients with PsA [149,150]. As in RA and SLE, it is probable that engagement of TLRs activates inflammatory cells and promotes secretion of pro-inflammatory cytokines, such as IL-6, TNFa, and type I IFNs, which not only drive inflammation in AS, but also could further activate macrophages in the atherosclerotic plaques and thus promote atherogenesis.

Recently, there is much interest in the possible role of NOD-like receptor family pyrin domain containing 3 (NLRP3) inflammasome in atherosclerosis. The NLRP3 inflammasome is an intracellular signaling molecule that, after being stimulated by TLRs and NFkB, activates caspase-1, which in turn cleaves the pro-inflammatory cytokines IL-1β and IL-18 to their active forms, known to play a vital role in promoting the development of lipid plaques and destabilizing the plaques [151,152]. In a study by Altaf et al. NLRP3 and downstream cytokine expression were increased in the peripheral blood of patients with acute MI and unstable angina compared to controls and this was modulated by high dose rosuvastatin [153]. High expression of NLRP3 was also evident in carotid atherosclerotic plaques and this was associated with plaque vulnerability [154]. The implication of NLRP3 in atherogenesis is further supported by the fact that the inhibition of NLRP3 reduced plaque formation and decelerate atherosclerosis in mice [155]. Studies have shown NLRP3 is activated by cholesterol crystals and releases IL-1β [156,157], also demonstrating that the deposition of crystalline cholesterol in arteries or elsewhere is an early cause rather than a late consequence of inflammation.

NLRP3 inflammasome, being an important component of the inflammatory process, has also been shown to have major involvement in the development of CIRD. Several studies have implicated the activation of NLRP3 and subsequent IL-1β secretion in the pathogenesis of RA, as NLRP3 gene expression and caspase-1 and IL-1β levels were elevated in patients with active RA [158,159]. Finally, a study by Kastbom et al. showed that Genetic variants of the NLRP3 inflammasome increased the risk of stroke/transient ischemic attack in RA patients [160]. In patients with SLE, studies have shown elevated circulating IL-18, IL-1β and IL-17, as well as hyperactive NLRP3 inflammasome and caspase-1 in peripheral blood monocyte cells and lupus nephritis biopsies, and this was correlated with serum levels of anti-dsDNA antibodies and disease activity and even linked to lupus nephritis [161,162]. Immune complexes formed secondary to antibody recognition of DNA or RNA antigens and C3a have been shown to stimulate inflammasome activation through the upregulation of TLRs and upregulation of ATP secretion, respectively [163,164]. Regarding the association with CVD, type I IFNs present in SLE are considered to have a detrimental impact on the vasculature that promotes atherosclerosis, and this is done by upregulation of NLRP3, caspase-1, and IL-18 in endothelial progenitor cells [165]. Moreover, Samstad et al. recently suggested that complement activation may contribute to atherosclerosis development via enhancement of NLRP3 inflammasome by cholesterol crystals, which accumulate in atherosclerotic plaques [166]. In AS, data is scarce and contradictory concerning the role of NLRP3 inflammasome in pathogenesis, as Zhao et al. reported association with AS susceptibility [167], whereas Kastbom A et al. found no association between them [160].

#### 4.1.3. Macrophage Accumulation

Macrophages are fundamental contributors in the development and progression of atherosclerosis. Atherosclerosis begins with a fatty streak, which is made up almost entirely of monocyte-derived macrophages. Macrophages and monocytes ingest oxidized LDL, transform into foam cells, and recruit additional monocytes and macrophages to the vessel wall. The development of an atheroma continues as other inflammatory cells are recruited to the intima and as smooth muscle cells proliferate, thereby increasing the lesion size [28,168].

The accumulation of macrophages at the site of inflammation producing inflammatory mediators serve as a prominent feature in both systemic inflammation and atherosclerosis. In both RA and SLE macrophage activation, as reflected by serum neopterin concentrations [169,170], is increased compared to controls. This increase was more robustly associated with mediators of inflammation in SLE rather than RA, however, this could be attributed to IFN-γ, which induces neopterin formation and is known to be implicated in the pathogenesis of SLE. In the same study, the lower concentrations of neopterin found in patients with SLE taking antimalarials and in those with RA taking MTX suggest that these drugs may inhibit monocyte and macrophage activation [171]. In another study by Voloshyna et al. [172], the presence of RA plasma induced pro-atherogenic changes in gene expression and was associated with augmented lipid accumulation and foam cell formation by macrophages, suggesting that chronic exposure to RA plasma adversely affects the capacity of monocytes/macrophages in the arterial wall to metabolize cholesterol and maintain lipid homeostasis, thereby contributing to the development of premature atherosclerosis.

In SLE, priming of IFNα, which plays a pathogenic role in the disease, promotes lipid uptake and macrophage-derived foam cell formation, indicating that the IFN signaling pathway may be linked to the risk of atherosclerosis by affecting plaque formation in patients with SLE [173]. Moreover, recent data show a different regulation of gene expression in mononuclear cells during monocyte-to-macrophage differentiation in SLE patients compared with controls and patients with atherosclerosis, thus suggesting a common pathogenic base between SLE and atherogenesis [174]. We were not able to retrieve studies specifically examining the role of macrophage accumulation and activation and promotion of atherosclerosis in AS or PsA, however, pathogenic similarities between atherosclerosis and psoriasis, involving monocyte/macrophage infiltration and secretion of chemokines and cytokines, could also stand for PsA [175].

#### 4.1.4. Pro-Inflammatory Cytokines

Proinflammatory cytokines are involved in all stages of atherosclerosis and therefore are considered key mediators in the pathogenesis of CVD. From activation of the endothelium and recruitment of immune cells to monocyte differentiation, foam cell formation, plaque rupture, and thrombosis, cytokines orchestrate the whole inflammatory process; both the innate and adaptive immune response [176,177].

Dyslipidemia is promoted by sustained inflammation as certain cytokines, namely TNFa and IL6, have been shown to influence lipid levels, shifting them towards an atherogenic profile [178]. Other cytokines, such as IFN-γ and TNF-α can modulate the permeability of vascular endothelial cells to macromolecules such as LDL and activated endothelial cells release chemokines and other cytokines in order to recruit immune cells, particularly monocytes and T-lymphocytes, in the lesion [179].

Cytokines are also responsible for the differentiation of monocytes to macrophages in the arterial intima and the macrophage differentiation to phenotypes. T-helper-1 (Th1) cytokines such as IFN-γ and IL-1β favor M1 phenotype, which produces pro-inflammatory cytokines such as IL-6, IL-12 and TNF-α, whereas Th2 cytokines such as IL-4 and IL-13 are required for M2 phenotype, which produces anti-inflammatory cytokines such as IL-10 and TGF-β. TNF-a, IL-4, and IL-13 are also known to promote LDL oxidation by monocytes/macrophages and IFN-γ stimulates macrophage foam cell formation by increasing the uptake of modified LDL and decreasing cholesterol efflux. A recent review highlighted the cellular and oxidative mechanisms of IL-6 signaling in the vasculature, including endothelial activation, vascular permeability, immune cell recruitment, endothelial dysfunction, as well as vascular hypertrophy and fibrosis [180,181].

TL1A is another inflammatory cytokine and member of the TNF superfamily of ligands, whose impact on atherogenesis has recently been a topic of interest. TL1A, via binding to death-domain receptor 3 (DR3) expressed in lymphocytes, is important for T-cell costimulation and Th1 polarization, the induction of pro-inflammatory cytokines/chemokines, and also promotes macrophage foam cell formation [182,183]. Furthermore, various growth factors produced by macrophages, such as ECs and T-cells, control the migration and proliferation of smooth muscle cells and other pro-inflammatory cytokines, such as IFN-γ and TNFa, affect plaque vulnerability by stimulating the apoptosis of macrophages and of smooth muscle cells. Finally, certain pro-inflammatory cytokines, such as TNF-a and IL-6, also suppress natural anticoagulant mechanisms, such as the protein C pathway, therefore favoring coagulation [178].

As in atherosclerosis, it is well known that aberrations in cytokine expression and signaling have pivotal pathogenetic roles in CIRD. In RA, many of the local and systemic manifestations appear to result from the production of a variety of cytokines within the inflamed synovium, particularly TNF-α, IL-1 and IL-6 [184], and inhibition of these cytokines with biologic agents is currently a main therapeutic option for patients with RA. Interestingly, between the proinflammatory cytokines involved in the pathogenesis of RA, TNFa and IL6 are independently predictive of a subsequent CV event, suggesting a more direct effect of these cytokines on the endothelium [185]. The overexpression of these proinflammatory cytokinesin RA could be responsible for the accelerated CVD risk. Increased production of IFNγ in RA, as a result of a significant amount of CD4^+^CD28^−^ cells that promoteTh1cell activation, could also have a critical role in accelerated atherosclerosis [186,187].

Regarding TL1A, recent studies have demonstrated that this cytokine is overexpressed in synovial fluids and synovial tissue, as well as serum of rheumatoid factor (RF)-seropositive RA patients, and this expression is correlated with disease activity [188,189]. In addition, individual serum levels of TL1A correlated with the progression of carotid atheromatic plaque height and the formation of new plaques, indicating that the dysregulated TL1A induced signaling may be associated with risk for accelerated atherosclerosis in RA [190].

Finally, cytokine-induced metabolic effects could induce atherosclerosis by altering the classical CVD risk factors. Cytokines such as TNFa and IL-6 favor insulin resistance in RA patients, as well as a more atherogenic lipid profile [83,103,191]. This is amplified by the fact that anti-TNF treatment improves insulin resistance and increases the levels or atheroprotective HDL, however, it does not seem to ameliorate the overall atherogenic index in the long term, while anti-IL6 treatment seems to improve insulin resistance but increases levels of total cholesterols, LDL, and triglycerides [89,192].

The link between SLE pathogenesis and atheromatosis mainly concerns the innate immune system rather than T and B cells. More specifically, IFN-γ, which is well known to play a central role in SLE pathogenesis, is also implicated in the promotion of atherosclerosis, as the majority of pathogenic T cells are of the Th1 profile, producing high levels of IFN-γ. IFN-γ activates monocytes/macrophages and dendritic cells, leading to the perpetuation of the pathogenic Th1 response that promotes smooth muscle cell proliferation. Moreover, type I interferons, that have also been postulated to play a central role in SLE pathogenesis [193], constitute a critical link between the two diseases. IFN-α increases the uptake of oxLDL and enhances foam cell formation and IFN-β affect the adhesion and migration of leucocytes to plaques and promotes plaque rupture, possibly attributing the higher prevalence of CV events in SLE to plaque instability [176,194,195]. As previously mentioned, TNFa and IL-6 have been shown to influence lipid levels in SLE, shifting them towards an atherogenic profile [104,178,194].

Data on the pathogenic role of cytokines and atherosclerosis in AS and PsA is scarce. Among pro-inflammatory cytokines, TNF-α is a key pathogenic factor in AS and IL-17 and IL-23 in PsA. As in RA, TNFa is implicated in the pathogenesis of atherosclerosis and may also have a harmful effect on lipid profile in patients with AS. Regarding IL17 and IL23, data from different studies are contradictory, as IL-17 has been thought to be atheroprotective as well as proatherogenic. IL-17 seems to inhibit the development of Th1 cells by activation of IL-17 receptors. On the other hand, higher IL-17 expression in human carotid plaques was associated with a more stable phenotype. Apart from the direct effect on the vasculature, IL17 and IL-23 are known to be elevated in obese individuals and are implicated in the development of MetS, as they increase IR [21,196,197].

## 5. What It All Means for Treatment of Patients with CIRD

The therapeutic effect of different biologic and non-biologic antirheumatic drugs on CVD has been widely investigated in recent years. Firstly, antirheumatic therapies seem to differently affect classical CVD risk factors and MetS. In RA there is evidence from systematic reviews and large observational studies that MTX therapy may decrease CV morbidity and mortality [198]. The use of MTX is associated with a reduced chance of having MetS [87] but increases total cholesterol, LDL, HDL, and triglycerides in RA. However, these lipid increases are possibly due to the inflammatory-dampering effect and essentially reflect normalization of the lipid levels to those seen in the general population [90,91] and therefore are not generally believed to increase CV risk. We should point out that the pathophysiologic mechanisms causing increased prevalence of MetS differ between rheumatic diseases. For example, in SLE, insulin resistance was not associated with IL-6 or TNFa levels as in RA, only with BMI and ESR [103], therefore it is possible that each condition requires a more customized management for prevention of CVD.

On the other hand, established therapies classically used for the management of traditional risk factors, such as statins or angiotensin-converting-enzyme inhibitors, could have a favorable impact on CIRD by bearing an anti-inflammatory effect. A recent review by Soulaidopoulos et al. reported evidence on the angioprotective, antioxidative and anti-inflammatory properties of statins and their efficacy in reducing RA-disease activity, supporting that RA patients should be screened via non-invasive methods for increased subclinical CVD—namely by cIMT evaluation—in order to receive lipid-lowering therapy when needed [1,4,97].

Numerous studies have demonstrated that the use of biologic agents in CIRD blocking cytokines, such as TNF and IL6, can decelerate the atherogenic process and even improve endothelial function, arterial stiffness, or arterial wall hypertrophy. A recent meta-analysis focusing on the impact of TNFa inhibitors showed that TNF-a antagonist treatment induced a significant improvement in aortic PWV and Aix and therefore on CV risk [199]. The same stands for IL-6 receptor inhibition, as the use of the anti-IL-6 receptor antibody tocilizumab improved endothelial dysfunction and aortic stiffness in patients with RA [200]. A review by Tam et al. concluded that the use of biologic agents in RA probably is effective in preventing the progression of subclinical atherosclerosis and improving arterial stiffness, although results from different studies are contradictory, and it cannot be clear whether this effect goes beyond that of controlling inflammation irrespective of the disease modification strategy by which this is achieved [201]. Similarly, we have shown that effective disease control may abrogate any RA-specific effect on the progression of atherosclerosis, irrespective of treatment modalities [37].

In a large British cohort, patients with RA receiving TNF inhibitors have a decreased risk of MI compared with patients receiving cDMARDs [202]. Finally, a meta-analysis examining the association between CV events and antirheumatic drugs concluded that TNFa inhibitors and MTX are both associated with a reduced risk of CV events [203]. Data on other therapies is more limited. A recent review by Ketelhuth et al. found evidence from preclinical and clinical studies on the therapeutic effect of various immunomodulatory therapies on CVD, including IFNγ and IL17 [204]. Koga et al. argued that postnatal blocking of IFN-γ function by gene transfer of a soluble mutant of IFN-γ receptor in adult mice prevented the progression of established plaques that remodeled toward a more stable and less inflammatory phenotype [205], thus implying that a IFN-γ blockade could reduce CV events. Importantly, a recent secondary CVD prevention multicenter placebo-controlled study revealed that anti-inflammatory therapy targeting the interleukin-1β innate immunity pathway led to a significantly lower rate of recurrent CV events than placebo, independent of lipid-level lowering [206]. Such a claim paves the way for the use of biologic agents specifically for the management of CVD.

The use of biologic agents in the majority of studies was not associated with significantly increased infections compared to the placebo-treated group. However, the potential risk of the development of opportunistic infections due to immunosuppression should be noted, and the expected benefit from any biologic treatment should be considered in the context of this risk. 

Recently, the chemoattractant chemerin, which is considered an independent predictor of cardiovascular disease risk, was associated with endothelial activation and atherosclerosis in RA, and the blockade of TNFa and IL-6 seems to reduce chemerin concentrations in RA patients [207,208]. Similarly to classical risk factors, there is heterogeneity with respect to autoimmune-inflammatory risk factors between rheumatic diseases. Cytokines, such as TNF-α and immune complexes are primarily involved in arthritides, such as RA, AS and PsA, as well as in SLE. On the other hand, interferons and autoantibodies are rather involved in SLE-associated vascular conditions. IL17/IL23 are more involved in the pathogenesis of PsA. Therefore, the therapeutic management of CVD in each condition should be customized.

## 6. Practical Implications

The increased morbidity and mortality associated with CVD in CIRD designated the need for better understanding the common underlying pathogenic mechanisms. The common background of these conditions is inflammation and the dysregulation of the immune system, and numerous studies in recent years have focused on unraveling the exact process and molecules behind this interaction. Even though some of the studies performed present methological issues and therefore reach questionable and contentious results, the overall conclusion of an association between CIRD and accelerated atherosclerosis is definite. This way we could be able to effectively manage both atherosclerosis and CIRD with therapies targeting molecules and specific pathways that are implicated in the shared pathogenesis.

To ameliorate the impact of CVD in the morbidity and mortality of CIRD patients, it is important to promptly recognize and effectively manage classical modifiable CVD risk factors, as studies have shown that they are under-diagnosed and under-treated despite more close and regular monitoring [78,79]. As outlined above, inflammation alters the metabolic profile in these patients and effective treatment normalizes this metabolic state, however, anti-hypertensive treatment and especially lipid-lowering therapy should be initiated in each patient in such need early in the disease course. Since the decision to start a drug treatment of high blood pressure and lipids is based on total CV risk levels as assessed by international algorithms (e.g., the EU score), it is expected that the multiplication of these scores in (e.g., RA) will have a substantial impact on the management of CV risk factor in RA. Moreover, we should always take into account before initiation of therapies the demographic characteristics of each CIRD and each patient individually. For example, the age of disease onset for RA patients is usually greater than SLE or AS patients and AS patients are usually men, thus adding to the already increased CV burden. Interestingly, as previously mentioned, younger SLE women present higher relative risk of CVD compared to the general population than SLE women of more than 60 years of age. Lastly and more importantly, disease duration, apart from disease activity, seems to be an important factor determining CVD. This is more documented for RA [1,39] and several studies indicate that it applies for other CIRD.

We should point out that despite all the advances and research on the management of CVD in CIRD, studies still denote an increased prevalence of CV events and mortality in rheumatic diseases compared to the general population. More specifically, although the diagnosis and treatment of SLE has significantly improved and deaths due to lupus manifestations have decreased, those due to CVD in SLE have not; CVD remains a leading cause of death [209,210]. In PsA, two studies from the same cohort but from two different time periods demonstrated an increased overall mortality risk over nearly four decades of follow-up, but the mortality risk declined over time [211,212]. Finally, a recent meta-analysis by Mathieu et al. found a reduction in the prevalence and risk of MI and stroke in AS compared with a previous meta-analysis by the same researchers [18,109,213] probably attributable to improvements in the control of cardiovascular risk in AS.

The possible role of cytokine measurement in the diagnosis and prevention of CV events should be also considered. Wainstein et al. showed that IL-6 levels are predictive of significant coronary artery disease in patients referred for coronary angiography and Lin et al. concluded that IL-6 demonstrated a notable prognostic value for predicting cardiovascular mortality [214,215].

To conclude, CIRD and atherosclerosis share common pathogenic mechanisms and this raises the possibility of a common therapeutic strategy. Apart from the adequate management of cardiovascular comorbidities, which still remains insufficient, therapeutic options could include the blockade of proiflammatory cytokines implicated in both diseases. Whether the low relative risks of CVD in CIRD justify further research in the biomedical field to broaden therapeutic options remains open to debate. Future studies should focus on other potential targets implicated in atherosclerosis pathogenesis; i.e., the NLRP3 inflammasome or specific or multiple chemokines.

## Figures and Tables

**Table 1:** Cardiovascular mortality and morbidity, subclinical cardiovascular disease, and classical cardiovascular risk factors in RA, SLE, AS, and PsA.

		RA	SLE	AS	PsA
**CVD risk or mortality**		Comparative risk to DM [8,9,29] 1.5-fold risk compared to the general population [1] More than 1.5 risk of fatal CV event [31]	2- to 3-fold mortality (up to 16-fold) [12,13]	↑ vs. controls [32,33,34]	Prevalence resembles that of RA [35] ↑ mortality [32]
**CV events**	CHD	Similar risk as DM [10] 1.6–2.1 rate ratio for MI [17,30,31]	2- to 3-fold risk (up to 52- fold in young SLE women) [12,13]	1.4 relative risk of MI [18,36]	1.4 relative risk of MI [17]
	Stroke	1.9 rate ratio [31]	2-fold risk [12,13]	1.3-1.4 relative risk [18,36]	Similar or slightly increased prevalence [37]
**Subclinical CVD**	cIMT	↑ vs. controls [38]	↑ vs. controls [14,16]	↑ vs. controls [23]	↑ vs. controls [39]
	PWV	↑ vs. controls [40]	↑ vs. controls [41,42,43]	↑ vs. controls [44,45]	↑ vs. controls [46,47]
	FMD	↓ vs. controls [48,49]	↓ vs. controls [16]	↓ vs. controls [45]	↓ vs. controls [39]
	Aix	↑ vs. controls [40]	↑ vs. controls [50,51]	Similar to controls [23]	--
	Plaques	↑ carotid vs. controls [38] femoral analogous to DM [52]	2-fold risk [14,15,16], comparable to RA and DM [15]	Similar to controls [23]	3-fold risk vs. controls [39]
**Classical Risk factors**		↑ prevalence of HTN, which underdiagnosed and undertreated. ↑ IR, associated with disease activity TNFa and IL6 levels “Lipid Paradox” Oxidative changes to HDL structure ↓ physical activity smoking	↑ IR and MetS, associated with BMI and higher levels of inflammation. Compared to RA: - IR index is lower - IR is associated with obesity, not TNFa or IL6. - higher concentrations of Lp(a), associated with IR Pro-atherogenic lipids	↑ BP levels ↓ HDL and TC levels, probably associated with TNFa smoking male gender	worse metabolic profile than RA or AS ↑ BP levels impaired fasting glucose ↓ HDL levels ↑ TC levels central obesity Similar to RA and AS: association with disease activity and TNFa levels

CVD: cardiovascular, DM: diabetes mellitus, CHD: coronary heart disease, MI: myocardial infarction, cIMT: coronary intima-media thickness, PWV: pulse wave velocity, FMD: flow mediated vasodilation, Aix: augmentation index, HTN: hypertension, IR: insulin resistance TNFa: tumor necrosis factor a, IL6: interleukin 6, HDL: high density lipoprotein, MetS: metabolic syndrome, BMI: body mass index, Lp(a): lipoprotein a, TC: triglycerides, vs.: versus. ↑: increased, ↓: decreased.

## Abbreviation

ACPA	Anti-citrullinated protein/peptide antibodies
Aix	Augmentation index
APS	Antiphospholipid syndrome
AS	Ankylosing spondylitis
ASDAS	Ankylosing Spondylitis Disease Activity Score
BASDAI	Bath Ankylosing Spondylitis Disease Activity Index
BMI	Body mass index
cDMARDs	Conventional disease modifying antirheumatic drugs
CHD	Coronary heart disease
cIMT	Carotid intima-media thickness
CIRD	Chronic inflammatory rheumatic diseases
CRP	C-reactive protein
CV	Cardiovascular
CVD	Cardiovascular disease
DAMPs	Damage-associated molecular patterns
DM	Diabetes Mellitus
DR3	Death-domain receptor 3
ds-DNA	Double stranded deoxyribonucleic acid
e.g.,	For example
EC	Endothelial cell
ESR	Erythrocyte sedimantation rate
EULAR	European League Against Rheumatism
FMD	Flow-mediated vasodilation
HDL	High-density lipoprotein
i.e.,	Id est (that is)
ICAM	Intercellular adhension molecule
IFN	Interferon
IL	Interleukin
IR	Insulin resistance
LDL	Low-density lipoprotein
Lp(a)	Lipoprotein a
MetS	Metabolic syndrome
MI	Myocardial infarction
MTX	Methotrexate
NFkB	Nuclear Factor Kappa B
NLRP3	NOD-like receptor family pyrin domain containing 3
NO	Nitric oxide
NSAIDs	Non-steroid antiinflammatory drugs
oxLDL	Oxidised low-density lipoprotein
PAMPs	Pathogen-associated molecular patterns
PGI2	Prostacyclin
PRRs	Pattern-recognition receptors
PsA	Psoriatic arthritis
PWV	Pulse wave velocity
RA	Rhaumatoid arthritis
RNA	Ribonucleic acid
ROS	Reactive oxygen species
SLE	Systemic Lupus Erythematosus
SLEDAI	Systemic Lupus Erythematosus Disease Activity Index
SLICC	Systemic Lupus International Collaborating Clinics/American College of Rheumatology
SpA	Spondyloarthropathies
TC	Triglycerides
TGF	Transforming growth factor
Th	T-helper
TLR	Toll like receptor
TM	Thrombomodulin
TNFa	Tumor necrosis factor-a
VCAM	Vascular endothelial adhension molecule
